# Rediscovering the Axolotl as a Model for Thyroid Hormone Dependent Development

**DOI:** 10.3389/fendo.2019.00237

**Published:** 2019-04-12

**Authors:** Anne Crowner, Shivam Khatri, Dana Blichmann, S. Randal Voss

**Affiliations:** Department of Neuroscience, Spinal Cord and Brain Injury Research Center, and Ambystoma Genetic Stock Center, University of Kentucky, Lexington, KY, United States

**Keywords:** axolotl, paedomorphosis, metamorphosis, thyroid hormone, *ambystoma*

## Abstract

The Mexican axolotl (*Ambystoma mexicanum*) is an important model organism in biomedical research. Much current attention is focused on the axolotl's amazing ability to regenerate tissues and whole organs after injury. However, not forgotten is the axolotl's equally amazing ability to thwart aspects of tissue maturation and retain juvenile morphology into the adult phase of life. Unlike close tiger salamander relatives that undergo a thyroid hormone regulated metamorphosis, the axolotl does not typically undergo a metamorphosis. Instead, the axolotl exhibits a paedomorphic mode of development that enables a completely aquatic life cycle. The evolution of paedomorphosis allowed axolotls to exploit relatively permanent habitats in Mexico, and preadapted axolotls for domestication and laboratory study. In this perspective, we first introduce the axolotl and the various meanings of paedomorphosis, and then stress the need to move beyond endocrinology-guided approaches to understand the axolotl's hypothyroid state. With the recent completion of the axolotl genome assembly and established methods to manipulate gene functions, the axolotl is poised to provide new insights about paedomorphosis and the role of thyroid hormone in development and evolution.

## Introduction

Mexican axolotls (*Ambystoma mexicanum*) have been studied in laboratories throughout the world for over two-hundred years (1). Beginning in the early nineteenth century, French expeditions to Mexico brought preserved adult specimens back to Paris for examination by curators at the *Jardin des Plantes*. Esteemed zoologist Georges Cuvier originally classified these specimens as larvae of an unknown species (2). It was not until decades later, when living axolotls were brought to Paris and a laboratory population was established by Auguste Duméril, that these presumptive larval forms were found to be reproductively mature and capable of metamorphosis. From the same axolotl spawn, Duméril (3) observed that most sibs reached an adult state and some reproduced while retaining larval characteristics including external gills, while a few individuals metamorphosed into forms typical of terrestrial salamanders (4). The observation of both metamorphic and non-metamorphic forms arising from a single spawn inspired theories and experiments to explain the axolotl's unusual mode of paedomorphic development (5). While much has been learned from studies of the axolotl and other salamanders, the mechanistic basis of paedomorphosis remains largely unknown.

### Paedomorphosis

**Figure 1:**
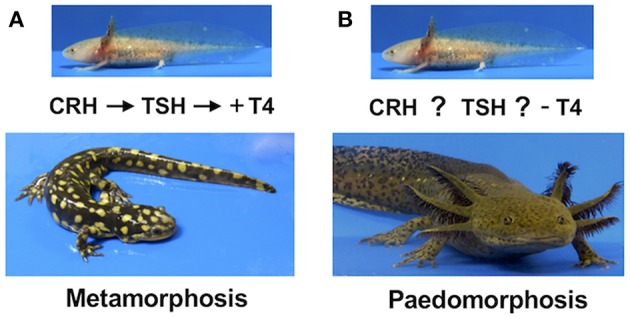
**(A)** The ancestral salamander mode of development is metamorphosis. Metamorphosis in the tiger salamander is regulated by the hypothalamus-pituitary-thyroid (HPT) axis. At a critical time during larval development, corticotrophin releasing hormone (CRH) from the hypothalamus stimulates thyrotrophic cells in the pituitary to release thyroid stimulating hormone (TSH), which in turn stimulates the thyroid gland to secrete thyroid hormone (TH). Increasing TH (+) triggers metamorphic changes in target cells. **(B)** The derived paedomorphic mode of development. Paedomorphosis in the axolotl results in the retention of ancestral larval characters into the adult phase of life. Although CRH-like and TSH-like activity are present in the axolotl hypothalamus and pituitary respectively, and TSH and TH treatment can induce metamorphosis, the HPT axis is not activated during larval development. Modified from Johnson and Voss (7).

Paedomorphosis is a somewhat confusing term because it has been used to explain variation at evolutionary, ecological, and genetic levels of inquiry. At an evolutionary level, paedomorphosis is used to describe a specific pattern of developmental variation among ancestral and descendant species (6). The ancestral mode of development in salamanders is generally thought to include a single, obligate metamorphosis which partitions the life cycle between an early aquatic phase and a more terrestrial adult phase. Indeed, close tiger salamander (*A. tigrinum*) relatives of the axolotl are known to invariably undergo a metamorphosis (Figure 1). In contrast, the axolotl typically does not undergo a metamorphosis; axolotls are paedomorphic because they express ancestral juvenile traits in the adult stage of life. Paedomorphosis thus provides an evolutionary explanation for how new patterns of variation arise among species; the biphasic life cycle of an ancestral species was truncated somehow during evolution to yield a paedomorphic species. Within the lexicon of heterochrony, a theory that associates changes in developmental timing to the origin of new forms, Gould (6) proposed that paedomorphic salamanders arose during evolution as a result of changes in mechanisms that regulate metamorphic timing. While such description is useful for describing evolutionary patterns of developmental variation, what we ultimately seek is proximate-level understanding of timing mechanisms that regulate the expression of paedomorphosis.

In ecological studies, paedomorphosis is used to describe patterns of developmental variation among individuals within species and populations. At this level, paedomorphosis is thought to be an adaptive developmental strategy for exploiting favorable larval habitats for growth and reproduction (8), although non-adaptive variation in the expression of paedomorphosis is known (9). The mechanisms that allow for such plasticity are presumably influenced by an individual's genetic makeup, health status, and environmental cues. For example, studies of salamanders that express paedomorphosis facultatively have shown clear ecological correlates. Paedomorphosis is more frequent in permanent aquatic habitats that do not undergo seasonal drying (10, 11). It has been argued that ecological conditions largely dictate the expression of metamorphosis or paedomorphosis in facultative species to increase fitness-associated traits, such as body size (bigger is generally better) and the probability of earlier and more frequent reproduction (12). It is important to point out that genetics also plays a role. Evidence for a population genetic component of variation has been shown in experiments that altered the heritability of paedomorphosis in *A. talpoideum* over several generations of selection (10). However, such studies do not provide resolution of genetic factors that regulate the expression of paedomorphosis.

Paedomorphosis has also been used to describe developmental variation among siblings within genetic crosses. Taking advantage of the recent evolution of paedomorphosis among tiger salamander complex species, interspecific genetic crosses have been performed in the laboratory to segregate metamorphic and paedomorphic modes of development and map the genomic location of genetic factors (13–18). These crosses have identified major effect quantitative trait loci (QTL) that regulate the timing of metamorphosis and expression of paedomorphosis. For example, second generation backcross individuals of *A. mexicanum* x *A. tigrinum* hybrid crosses that inherit axolotl alleles at the *met1* QTL delay metamorphosis or express paedomorphosis. It is not clear if genetic factors identified from interspecific crosses regulate the expression of metamorphosis and paedomorphosis in natural populations. However, this classical genetic approach presents an unbiased method to identify candidate genes and associated mechanisms that may be operative in natural populations.

### Endocrinology of Paedomorphosis

Over the last century, axolotl paedomorphosis has been the subject of a number of physiological studies. We refer readers to two relatively recent reviews of the literature pertaining to the endocrinology of axolotl paedomorphosis (7, 19). Our goal here is to briefly review salient features of axolotl hypothyroidism to provide context for identifying mechanisms that may regulate metamorphic timing and expression of paedomorphosis.

Thyroid hormones play a central role in regulating amphibian metamorphosis (Figure 1). Increasing titers of thyroid hormone during larval development are associated with tissue-specific changes that occur during metamorphosis. Thus, the timing of metamorphosis is potentially associated with a number of mechanisms that regulate TH: (1) the hypothalamus-pituitary-axis that regulates TH synthesis and secretion from the thyroid gland, (2) TH transport and uptake within cells, (3) activation and inactivation of TH within cells, (4) binding of TH to steroid nuclear receptors in the nucleus, and (5) the interaction of TH and TR with protein complexes that regulate transcription. TH levels in the axolotl remain low throughout larval development and do not increase at the time metamorphosis occurs in related tiger salamanders. However, the axolotl is capable of initiating and completing metamorphosis when thyroid hormone and other endocrine factors of the HPT axis are administered. Collectively, these and other results suggest a defect in the regulation of the HPT axis, perhaps at the level of the hypothalamus or pituitary (19–24). Given the importance of thyroid hormone feedback on HPT axis maturation and control, axolotl paedomorphosis presents a conundrum—are TH levels inherently too low to support normal development and function of the hypothalamus and pituitary, and/or are these glands relatively insensitive to TH feedback?

One mechanism that has been advanced to explain axolotl paedomorphosis is a failure in hypothalamic stimulation of pituitary thyrotropes that secrete thyrotrophin (TSH) to regulate thyroid activity (19). In contrast to mammals, corticotropin (CRH) and not thyrotropin releasing hormone (TRH) mediates release of TSH from the larval amphibian pituitary (25). CRH treatment of metamorphic tiger salamanders decreases the time to metamorphosis (26), consistent with thyrotrophic stimulation, while CRH treatment does not increase circulating T4 levels in the axolotl (24). While this result is consistent with a failure in hypothalamic stimulation of the pituitary, CRHR2 expression in axolotl thyrotropes appears to be normal. This highlights critical knowledge gaps in our understanding of axolotl paedomorphosis. Studies that have interrogated aspects of endocrine regulation have not rigorously controlled axolotl age, body size, or sex. Inducing metamorphosis by TH treatment of adult axolotls ignores early larval developmental windows within which the HPT axis matures and becomes operative. Also, studies have not assessed mechanisms of thyroid hormone regulation within all HPT axis tissues. For example, while it is clear that axolotls have functioning thyroid hormone receptors (27), the expression of TRs in HPT axis tissues has not been assessed. Also, while CRH is clearly essential for releasing TSH during amphibian metamorphosis, the secretion of CRH or CRH-like peptides has not been shown in the axolotl (19). Finally, much of what we know about the endocrinology of amphibian metamorphosis comes from studies of anurans, not from studies of salamanders. These two amphibian groups diverged several hundreds of millions of years ago and salamander families are minimally 150 million years diverged. Thus, it is not clear that the anuran metamorphic knowledge base, or the relatively fewer insights gained among salamander species, can provide a framework to conceptualize the HPT axis in axolotls. While endocrinology-guided approaches have provided important insights, there is need to consider other avenues of reasoning in the study of axolotl paedomorphosis.

### Genetics of Paedomorphosis

As was introduced above, genetic studies are beginning to resolve the location of genetic factors within the axolotl genome that regulate metamorphic timing and expression of paedomorphosis. Primarily, axolotls have been crossed to metamorphic tiger salamanders to segregate alleles that affect paedomorph expression and metamorphic timing, however we highlight a study (17) that crossed the axolotl to a paedomorphic relative to identify genetic factors associated with T4 sensitivity. In that study, second generation *A. mexicanum/A. andersoni* paedomorphic hybrids were created and administered 50 mM T4 at the time metamorphosis occurs in metamorphic tiger salamanders. Siblings exhibited tremendous variation in metamorphic timing (160-day range) and some individuals remained paedomorphic after 150 days of continuous T4 treatment. Genetic linkage mapping was then used to identify three moderate effect QTL (*met1-*3) that additively explained variation in metamorphic timing, including *met1* first identified in *A. mexicanum/A. tigrinum* hybrids. This study showed that metamorphic timing is associated with QTL that segregate allelic variation for responsiveness to T4.

At the time these QTL were identified and in lieu of a sequenced axolotl genome, comparative genome mapping was used to identify candidate genes for *met1-*3. The expressed sequence tag (EST) that was used to initially map *met1* showed similarity to *nerve growth factor receptor* (*ngfr*). As more genes were mapped, it became clear that this *ngfr-like* gene corresponded to *nradd* and *met1* located to a genomic region that was uniquely structured during vertebrate evolution. The genes in this region are found on separate chromosomes in all other vertebrates, including the newt (*Notophthalamus viridescens*) which is member of a different salamander family (28). Page et al. (18) speculated that this chromosomal fusion may have brought genes into linkage that are relevant for paedomorph expression in *Ambystoma*, because several of the genes have neurological functions. In addition to *nradd*, which is expressed in the mouse hypothalamus (29), *ccm2, map2k3*, and genes from the Smith-Magenis syndrome region in the human genome associate with *met1. ccm2* is associated with neurovascularization (30) while *map2k3* was recently identified as a superior memory candidate gene in SuperAgers (31). Smith-Magenis syndrome is primarily attributed to deletion polymorphisms of *rai1* (32), a dosage-sensitive transcription factor that regulates multiple functions, including embryonic neurodevelopment, neuronal differentiation, and circadian rhythm. Although *rai* locates outside the *met1* region, it is possible that long range enhancers for *rai1* or other flanking genes may locate within the *met1* region (33). While no candidate genes were identified for *met2, met3* is associated with *pou1f1*, a transcription factor associated with combined pituitary hormone deficiency in humans. This deficiency is associated with incomplete secretion of pituitary hormones involved in regulating growth and development, but not reproduction. This makes *pou1f1* a good candidate for metamorphic regulation because metamorphic and paedomorphic ambystomatids do not show differences in reproductive potential, although paedomorphic species can reproduce multiple times annually while metamorphic species breed once annually (7).

### New Axolotl Genome Resources

Five years ago, it was difficult if not impossible to pursue studies of axolotl candidate genes identified by comparative mapping. At that time, the large axolotl genome (32 Gb) had not been sequenced and thus it was difficult to develop molecular probes and investigate gene functions. However, in just the past couple of years, the axolotl genome has been incrementally sequenced and recently a chromosome-level genome assembly was completed (34). Now it is possible to comprehensively evaluate *met1-3* genomic regions for candidate genes and test gene functions using genome-editing approaches and transgenics. In this section, we review new, critical resources to identify and test candidate genes for future studies of axolotl paedomorphosis.

**Figure 2:**
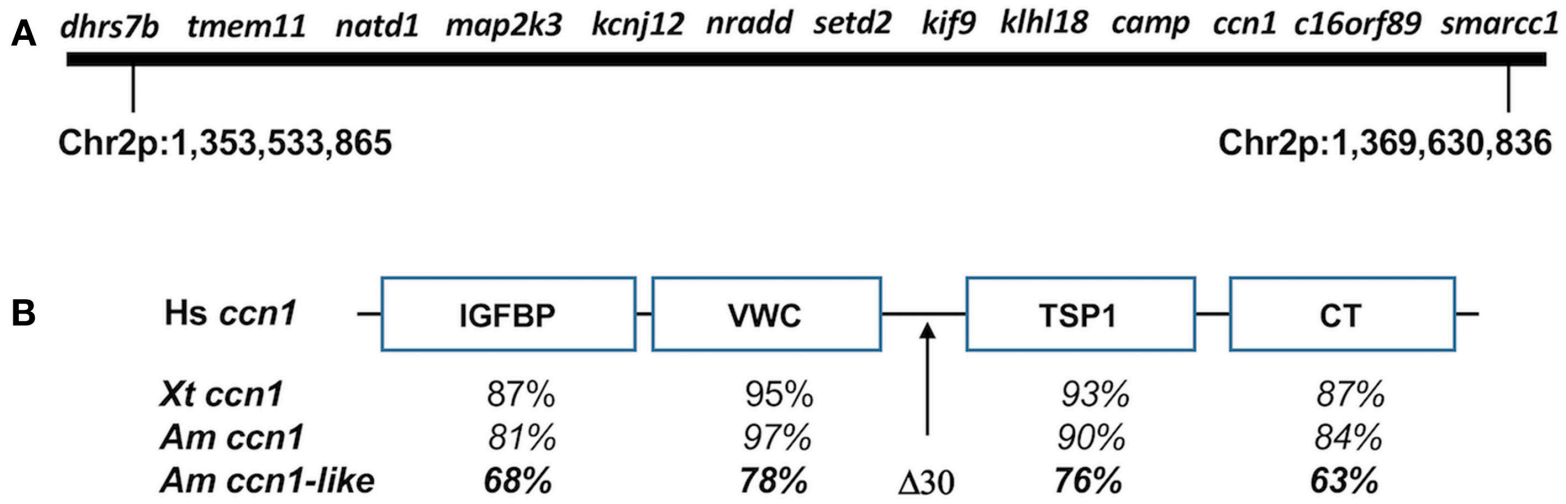
**(A)** Map of genes from axolotl chromosome 2 (Chr2p) that associate with *met1*. **(B)** General structure of CCN family proteins showing insulin-like growth factor binding protein-like (IGFBP), a von Willebrand factor type C repeat (VWC), thrombospondin-homology type 1 repeat (TSP1), and a C-terminal cysteine-knot-containing (CT) domains. Human (Hs) *ccn1* (GenBank: AAB84227.1) was used as a reference to compare domain-specific amino acid similarity among *Xenopus tropicalis* (Xp) *ccn1* (GenBank: OCA36969.1), *A*. *mexicanum ccn1* (AMEXTC_0340000257024_cysteine-rich), and *A. mexicanum ccn1-like* (AMEXTC_0340000025590_LOC102363594). The *A. mexicanum ccn1-like* sequence has a unique deletion of 30 amino acids between the VWC and TSP1 domains.

It is important to note that the method used to assembly the axolotl genome simultaneously increased the resolution of candidate genes within the *met1* genomic region (34). Forty-eight individuals from the meiotic mapping panel that was used to map *met1* (16) were sequenced to 2x depth to identify polymorphisms that in turn were used to order genomic scaffolds into chromosomes by linkage analysis. The 48 individuals that were sequenced were not randomly drawn from the mapping panel. Instead, individuals that exhibited recombination within the *met1* genomic region were purposely chosen for sequencing to more finely resolve the boundaries of recombination that differentiate early (metamorphic) vs. late (paedomorphic) metamorphosing individuals. Inspection of these recombination boundaries against the background of the physically ordered loci yielded a high confidence genomic region of relatively few candidate genes (Figure 2).

The ordering of *map2k3-nradd-and setd2* is the same in the genome assembly as it was determined previously by genetic linkage analysis. Page et al. (18) mapped these loci near the maximum inflection point of the *met* 1 LOD peak, implicating them as especially good candidate genes. The new genome assembly revealed new, physically-linked candidates, including *dhrs7b, tmem11*, and *natd1* from the Smith-Magenis syndrome region, and *kcnj12, klhl18, smarcc1*, and three anonymous, predicted genes. Using BLASTn and BLASTx searchers of the anonymous genes against NCBI databases, we discovered the likely identity of all three loci: *camp* (LOC101951429), *ccn1-like* (LOC102363594), and *c16orf89* (LOC102943813). While *camp* is known to be syntenic with *setd2, kif9*, and *khlh18* in the human genome, *ccn1-like* and *c16orf89* are not expected to map to the *met1* region, which only contains loci from human chromosomes 3 and 17. *ccn1* is a member of the CCN gene family, which contains six different members (CCN1-6). CCN family members typically have an N-terminal secretory signal peptide and four structural domains: an insulin-like growth factor binding protein-like domain, a von Willebrand factor type C repeat (VWC) domain, a thrombospondin-homology type 1 repeat (TSP1) domain, and a C-terminal cysteine-knot-containing (CT) domain. Interestingly, the *met1 ccn1-like* gene shows sequence similarity to *ccn1* but has a 30 bp deletion in the hinge region between the VWC and TSP1 domains (Figure 2). We determined the location of each presumptive CCN ortholog in the axolotl genome assembly, including a second *ccn1-like* gene that showed higher sequence identity to *ccn1*-vertebrate orthologs. The position of the second *ccn1-like* gene in the axolotl genome assembly suggests it to be the true vertebrate *ccn1* ortholog and thus the *ccn1*-*like* gene in the *met1* region appears to represent a novel gene. An ancient origin for this novel gene seems likely because it is equally divergent in amino acid similarity from human, *Xenopus tropicalis*, and axolotl *ccn1* orthologs (Figure 2). In contrast, the positioning of *c16orf89* in the met genome region likely reflects an intrachromosomal inversion as several genes linked to *c16orf89* in the human genome are found on axolotl chromosome 2, although some distance away.

### How to Test the Candidate Genes for Paedomorphosis?

It is important to point out that none of the candidate genes in the *met1* region have been annotated to endocrine gland development or endocrine process gene ontologies (Table 1). Two genes (*setd2, smarcc1*) that regulate global patterns of transcription during early neural development in mammals maybe interesting candidates to pursue because brain transcriptional activity is generally lower in larval axolotls than larval tiger salamanders (18, 35). Thyroid hormone and NGF signaling (perhaps via *nradd*) mediate early neuronal development in mammals, and based upon transcriptional data, *c16orf89* is predicted to play a role in thyroid gland development and function. Given the multifunctional roles of CCN family members, *ccn1-like* presents an attractive candidate. However, nothing is known about this genes function and none of the genes discussed above seem to standout above the rest. As a path forward, we believe that all of the genes in the *met1* region can be quickly tested for function using CRISPR-Cas9, which is known to efficiently knock-out genes in the axolotl. For example, CRISPR-Cas9 was used recently to create insertion/deletion polymorphisms in *fat3* to generate offspring that presented limb and kidney defect phenotypes seen in the *short toes* axolotl mutant (36). In a similar vein, CRISPR-Cas9 could be used as a strategy to test candidate genes in the *met1* genomic region using offspring from axolotl x tiger salamander crosses. For example, it is well-established that all axolotl x tiger salamander F1 hybrids undergo metamorphosis. This suggests that dominant tiger salamander alleles at candidate genes are sufficient to induce metamorphosis. Conversely, this suggests that paedomorphosis is associated with the inheritance of recessive alleles that may be partially functional or null in regards to inducing metamorphosis. To test this hypothesis, it would be efficient to knockout candidate genes in F1 hybrids as only the tiger salamander allele would need to be edited. F1 individuals could be assayed for paedomorphosis or delayed metamorphic timing and thus in short order, each of the candidate genes could be tested functionally. We note that it would also be informative to knock out *met1-3* candidate genes in other vertebrates, including frogs which do not present paedomorphosis.

**Table 1:** Candidate genes from the *met1* region with functional information obtained from the NCBI Gene database.

**Gene ID**	**Gene information**
*dhrs7b*	Short-chain dehydrogenase/reductase family member. Possibly could function in steroid hormone regulation.
*tmem11*	Mitochondrial inner-membrane protein thought to regulate mitochondrial morphogenesis.
*natd1*	The function of this gene is unknown but transcripts in mice embryonic stem cells suggest a role in hematopoiesis.
*map2k3*	MAP kinase-mediated signaling cascade member that activates MAPK14/p38-MAPK in response to mitogens and environmental stress.
*kcnj12*	Inwardly rectifying K+ channel generally associated with heart function although broadly expressed among other tissues in human.
*nradd*	Highly similarity to the p75 neurotrophin receptor *ngfr* that functions in neurotrophin signaling in rodents.
*setd2*	Histone methyltransferase that is specific to lysine-36 of histone H3. Methylation of this residue is associated with active chromatin. Interacts with histone H2A.z to regulate embryonic neurogenesis in mice.
*kif9*	Kinesin motor protein that functions in the regulation of spindle length and chromosome alignment during mitosis.
*klhl18*	Associates with *cul3* ubiquitin ligase to regulate cell cycle entry.
*camp*	An antimicrobial and immune response protein.
*ccn1-like*	Novel CCN family gene. CCN family proteins regulate many cellular responses that are critical for skeletal, vascular, and neural development.
*c16orf89*	The function of this gene is unknown but human transcripts are enriched in the thyroid gland.
*smarcc1*	Important component of the large ATP-dependent chromatin remodeling complex SNF/SWI which functions during brain development to regulate transcription globally.

## Conclusion

Although the Mexican axolotl has been studied for over 150 years, the mechanism associated with its unique paedomorphic mode of development remains unknown. Endocrinology studies have established the importance of thyroid hormone in regulating amphibian metamorphosis. The axolotl does not show an increase in thyroid hormone during early development and thus fails to undergo metamorphosis. While many aspects of the HPT axis seem to be functional in the axolotl, and peripheral tissues are responsive to thyroid hormone treatment, endocrinology-guided studies have not resolved the basis of paedomorphosis. We argue the need to test candidate genes from genetic studies of axolotl paedomorphosis using new genomic resources available to the community. In particular, the new axolotl genome assembly has resolved a short-list of candidate genes for the *met1* genomic region that can be efficiently tested using CRISPR-Cas9 to knock-out gene functions. The recent development of essential genetic and genomic tools for the axolotl brings us closer to identifying mechanisms of paedomorphic development and understanding the role of thyroid hormone in development and evolution.

## Author Contributions

AC, SK, DB, and SRV collectively wrote the paper.

### Conflict of Interest Statement

The authors declare that the research was conducted in the absence of any commercial or financial relationships that could be construed as a potential conflict of interest.
